# Personal—and Unselfish

**Published:** 1867-10

**Authors:** 


### PERSONAL—AND UNSELFISH.

A YEAR ago, under a caption not much different from the above, we explained to the readers of the REGISTER that we were about to try the experiment of a medico-dental office. We then believed that every large city stood in need of such. We believe so still. The many ills resulting from, and those aggravated by dentition and its derangements, are too often misunderstood by the Dentist, and too often overlooked by the physician. In making this statement we say nothing against either profession. Our views on professional specialties are well known to the readers of this. Although very much of the severest suffering known to the human family results from Dental disease, yet the subject is often neglected by the general practitioner, on account of his time being taken up by morbid conditions more immediately fatal. Hence, we argued the propriety of Dental *Physicians*, as well as Dental *Surgeons*. But our experiment is a failure, in that it is not appreciated to a remunerative extent either by the profession or the public. Fortunately an improved state of health places the partial resumption of practical Dentistry within our power. We hope others will succeed where we have failed; and in resuming "chair-work," we are still willing to be useful to our professional brethren, either in the way of counsel, or in the use of anæsthetics. W.

